# Integrated GWAS and eQTL Colocalization Identified Candidate Genes for Growth Traits in Pigs

**DOI:** 10.3390/biology15141216

**Published:** 2026-07-22

**Authors:** Xiangzi Wu, Junjing Wu, Yiren Gu, Mu Qiao, Jiawei Zhou, Zipeng Li, Yue Feng, Tong Chen, Dake Chen, Shuqi Mei, Xianwen Peng, Zhong Xu

**Affiliations:** 1 Key Laboratory of Animal Embryo Engineering and Molecular Breeding of Hubei Province, Institute of Animal Sciences and Veterinary Medicine, Hubei Academy of Agricultural Sciences, Wuhan 430064, China; wxzi12580@163.com (X.W.); jeanne1106@126.com (J.W.); qiaomu@hbaas.com (M.Q.); zhoujiawei@hbaas.com (J.Z.); lizipeng@hbaas.com (Z.L.); fy914858517@163.com (Y.F.); ctokay@163.com (T.C.); chendake@hbaas.com (D.C.); meishuqi@hbaas.com (S.M.); 2 College of Animal & Veterinary Sciences, Southwest Minzu University, Chengdu 610041, China; guyiren1128@163.com; 3 Hubei Hongshan Laboratory, Wuhan 430064, China

**Keywords:** pig, growth traits, weighted GWAS, multitissue transcriptomics, candidate genes

## Abstract

Growth traits, as core economic indicators in pig breeding, are closely associated with production costs, rearing duration, and final carcass quality and have thus consistently been a major focus of genetic improvement. This study aimed to identify candidate genes affecting Age to 120 kg live weight (AGE120), Backfat thickness at 120 kg (BF120), and Loin muscle depth at 120 kg (LMD120) in pigs. Ear tissue samples were collected from 3364 healthy adult pigs (including 558 boars and 2805 sows) from three breeds: Large White, Landrace, and Duroc. Genotyping was performed using an 80 K functional site array, and quality-controlled SNP (Single-Nucleotide Polymorphism) loci were subjected to genotype imputation, resulting in 15,447,611 loci obtained. Genome-wide association studies (GWASs) for Age to 120 kg live weight, Back fat thickness at 120 kg, and Loin muscle depth at 120 kg were conducted using a mixed linear model in Genome-wide Complex Trait Analysis (GCTA). Genes located within 500 kb upstream and downstream of significant GWAS loci were extracted using the biomaRt package in R. Furthermore, colocalization analysis was performed using expression Quantitative Trait Locus (eQTL) data of 34 tissues from the PigGTEx database to identify genes that share the same causal variant as the GWAS signals. Through integrated GWAS and eQTL colocalization analysis, in addition to five previously reported genes associated with pig growth traits (*TAF11*, *ZC3HAV1L*, *ANKS1A*, *USP20*, and *TBC1D1*), a set of novel, high-confidence candidate genes was identified: *ZNF215*, *UBE2Z*, *HOXB7*, *SARDH*, *ADAMTSL2*, *ATP6V0A4*, *RPL10A*, *PGM2*, and *RELL1*. These findings enrich our understanding of the genetic architecture underlying growth traits in pigs at heavy body weights and provide an important foundation for subsequent functional validation and molecular breeding applications.

## Simple Summary

In pig production, growth traits such as the age to reach 120 kg body weight, backfat thickness, and loin muscle depth are economically important. Understanding the genetic factors that control these traits can help breeders select pigs that grow faster and have better carcass quality. In this study, we analyzed more than 3300 pigs from three commercial breeds (Large White, Landrace, and Duroc). We used genome-wide association studies (GWASs) to find genetic markers linked to the three growth traits and then combined these results with gene expression data (expression Quantitative Trait Loci, eQTL) from multiple tissues to identify the most likely causal genes. In addition to confirming five previously known genes, we discovered several new candidate genes that may influence growth through pathways such as protein turnover, muscle–fat balance, and metabolism. These findings provide valuable targets for improving pig growth efficiency and carcass traits through marker-assisted breeding or genomic selection.

## 1. Introduction

Growth traits, as core economic indicators in pig breeding, are closely associated with production costs, rearing duration, and final carcass quality and have thus consistently been a major focus of genetic improvement efforts [1]. In modern intensive farming systems, traits such as growth rate, backfat thickness, and degree of muscle development not only determine the finishing efficiency and feed conversion ratio of commercial pigs but also profoundly influence lean meat percentage and carcass cut-out value [2]. Parameters including Age to 120 kg live weight (AGE120), Backfat thickness at 120 kg (BF120), and Loin muscle depth at 120 kg (LMD120), owing to how they comprehensively reflect individual growth and development status as well as carcass composition characteristics, have become core measurement indicators for the genetic evaluation of breeding pigs worldwide.

Landrace, Yorkshire, and Duroc are the three most widely used lean-type pig breeds in the global swine industry. Among them, Yorkshire and Landrace are renowned for their excellent reproductive performance and adaptability and are commonly used as maternal lines in crossbreeding systems, while Duroc is widely employed as a terminal sire due to its fast growth rate, superior carcass quality, and strong stress resistance. Studies have shown significant interbreed differences among these three breeds in growth rate, backfat deposition patterns, and muscle development: Yorkshire outperforms Landrace and Duroc in average daily gain and feed conversion ratio, while Duroc exhibits significantly greater backfat thickness than Landrace and Yorkshire [3]. This genetic differentiation not only provides a basis for exploiting heterosis but also suggests that different breeds may possess distinct genetic growth-regulating mechanisms that warrant separate investigations. In recent years, with the advancement of swine genetic improvement efforts in China, the National Swine Genetic Improvement Plan (2021–2035) has designated AGE120, BF120, and LMD120 as core breeding traits. Consequently, the traditional evaluation system based on measurements at 100 kg body weight is gradually transitioning toward a heavier body weight standard.

Genome-wide association studies (GWASs) are powerful tools for dissecting the genetic basis of complex traits [4,5]. In recent years, researchers have used GWAS to identify a large number of genetic loci and candidate genes that are significantly associated with growth traits across multiple pig populations. In a population of 2360 Duroc pigs, researchers detected 32 significant SNPs (Single-Nucleotide Polymorphisms) associated with Age live weight (AGE), Average Daily Gain (ADG), Backfat Thickness (BF), and Loin Muscle Depth (LMD), and identified 46 potential candidate genes involved in muscle development, fat deposition, and cell growth regulation, including *CD55 NRIP1*, *TRIP11*, *MIS2*, *VRTN*, and *ZEB2* [2]. Current GWASs have mostly focused on single-breed populations, and most studies have used 100 kg body weight as the correction benchmark. A systematic comparison of the genetic architecture underlying growth traits at the 120 kg body weight level across different breeds is still lacking. More importantly, traditional GWASs can only localize genomic regions associated with traits, and most significant SNPs are located in noncoding regions. The mechanisms by which these SNPs influence target traits—whether by regulating the expression of nearby genes, acting through long-range chromatin interactions, or merely being in linkage disequilibrium with true causal variants—remain unresolved [6].

Colocalization analysis offers a critical breakthrough in this regard. This approach assesses whether GWAS signals and molecular quantitative trait locus signals within the same genomic region share the same causal variant, thereby linking associated loci to specific regulatory genes and functional tissues [7]. When a GWAS signal is detected to colocalize with an expression quantitative trait locus (eQTL), this suggests that the genetic variation in that region likely influences the phenotype by altering the expression level of a target gene. Recently, the strategy of integrating GWAS with eQTL colocalization analysis has demonstrated significant value in dissecting the genetic architecture of complex traits in pigs. Ayalew et al. [8] identified four potential causal genes for age at 100 kg (*ABCD4*, *PTGR2*, *ENTPD5*, and *FAM161B*) and two for backfat thickness (*EXOSC2* and *USP20*) in three commercial pig populations by combining GWAS with multitissue eQTL colocalization analysis using the PigGTEx project. In another study on Duroc pigs, a multiomics integration strategy (including eQTL colocalization, chromatin state annotation, and Hi-C interaction data) was employed to prioritize regulatory variants affecting stress resistance traits [9]. Furthermore, the PigGTEx project systematically assessed the colocalization of GWAS signals with eQTLs across 34 tissues, providing a valuable resource for linking complex trait-associated loci to effector genes [10]. These advances indicate that integrated GWAS and colocalization analysis has become a reliable approach to move from association signals to functional genes, representing a key strategy for transitioning from “localization” to “causality.”

Despite the important advances mentioned above, several scientific questions still need to be urgently addressed. First, the vast majority of current GWASs on pig growth traits are based on a corrected body weight of 100 kg, whereas the Chinese Swine Genetic Improvement Plan has explicitly set a body weight of 120 kg as the core selection stage. These two stages differ significantly in terms of growth and development patterns. During the process from 100 kg to 120 kg, the fat deposition rate increases markedly, while muscle deposition tends to plateau. Therefore, genetic findings based on the 100 kg benchmark may not be fully extrapolated to the 120 kg stage. Second, although Large White, Landrace, and Duroc—the three core breeds of the modern pig industry—exhibit significant differences in growth patterns and genetic backgrounds, joint GWAS analyses and comparative studies of growth traits at the 120 kg body weight level across these three breeds remain scarce. Third, although colocalization analysis has been widely applied to other species and traits, systematic studies integrating GWAS with multitissue eQTL colocalization in the context of pig growth traits are still limited. Most studies remain at the stage of simple localization, lacking in-depth identification of causal genes and tissue-specific regulatory mechanisms.

To address the aforementioned research gaps, in this study, we systematically collected phenotypic data on three growth traits—AGE120, BF120, and LMD120—in three lean-type pig breeds: Landrace, Large White, and Duroc. Based on breed-wise GWASs, we further integrated multitissue eQTL data from the PigGTEx project for colocalization analysis. We aimed to achieve the following objectives, namely to identify genetic loci and candidate genes significantly associated with growth traits at 120 kg within and across breeds; to identify the most likely causal genes and their tissues of action using colocalization analysis; and to compare the similarities and differences in the genetic architecture of growth traits among the three breeds, thereby providing theoretical foundations for breed-specific molecular breeding strategies. The findings of this study will deepen our understanding of the genetic regulatory mechanisms underlying growth traits in pigs at heavy body weights, provide important candidate gene resources for precision marker-assisted breeding and genomic selection, and ultimately contribute to the genetic improvement of lean-type breeding pigs in China.

## 2. Materials and Methods

### 2.1. Experimental Animals

In this study, a total of 3363 adult pigs (including 558 boars and 2805 sows) from a core breeding farm in Hubei Province were selected, comprising three breeds: Large White (1852 heads), Landrace (998 heads), and Duroc (513 heads). All pigs were healthy and in good condition, raised under identical feeding conditions and managed according to standard protocols.

### 2.2. Phenotypic Data

Basic information including individual ID, age in days, sex, and birth year was recorded for 3364 pigs. Traits such as birth weight, final test weight, days to 120 kg body weight, backfat thickness at 120 kg, and loin muscle thickness at 120 kg were measured. Statistical analyses were performed using R (v.x64 4.5.3) software to obtain the mean, minimum, maximum, standard deviation, and coefficient of variation for days to 120 kg body weight (AGE120), backfat thickness at 120 kg (BF120), and loin muscle depth at 120 kg (LMD120).

According to the requirements of the National Swine Genetic Improvement Plan (2021–2035), the core breeding traits for lean-type breeding pigs are defined as days to 120 kg, backfat thickness, and loin muscle depth, among others. Phenotypic data were collected and recorded for these traits and subsequently calculated using correction formulas.

Correction formula for days to 120 kg body weight in Table 1:

Days to target body weight = Actual age in days + (Target body weight − Actual body weight) × (Actual age in days − A)/Actual body weight

In the formula, the unit for days to target body weight is days; actual age in days excludes the day of birth, calculated by subtracting the birth date from the measurement date; body weight is measured in kilograms (kg). The term A in the formula is the correction constant, as provided in the table below.

**Table 1:** Correction formula for days to 120 kg body weight.

Breed	Recommended Value of Correction Constant A	
	Boar	Sow
Yorkshire	53.053	44.665
Landrace	54.863	47.834
Duroc	58.122	47.250

Correction formula for backfat thickness at 120 kg body weight in Table 2:

Backfat thickness at target body weight = Actual backfat thickness + (Target body weight − Actual body weight) × Actual backfat thickness/(Actual body weight − B)

In the formula, the unit of backfat thickness is millimeters (mm). The term B in the formula is the correction constant, as provided in the table below.

**Table 2:** Correction formula for backfat thickness at 120 kg body weight.

Breed	Recommended Value of Correction Constant B	
	Boar	Sow
Yorkshire	−9.975	−5.765
Landrace	−7.300	−1.146
Duroc	−10.811	−6.254

Correction formula for loin muscle depth at 120 kg body weight in Table 3:

Loin muscle depth at target body weight = Actual loin muscle depth + (Target body weight − Actual body weight) × (Actual loin muscle depth − C)/Actual body weight

In the formula, the unit of loin muscle depth is millimeters (mm). The term C in the formula is the correction constant, as provided in the table below.

**Table 3:** Correction formula for loin muscle depth at 120 kg body weight.

Breed	Recommended Value of Correction Constant C	
	Boar	Sow
Yorkshire	16.239	22.809
Landrace	24.413	25.729
Duroc	25.820	26.854

### 2.3. Genotype Data

Following the manufacturer’s instructions, genomic DNA was extracted from pig ear tissue using an Animal Tissue DNA Extraction Kit (Tiangen Biotech, Beijing, China; Catalog No. DP304). DNA concentration was measured using a Nano Drop 2000 spectrophotometer, and DNA quality was assessed by 1.5% agarose gel electrophoresis. DNA samples that passed quality control were submitted to Wuhan Yingzi Gene Technology Co., Ltd. (Wuhan, China) for 80 K functional site genotyping array analysis. Quality control was performed using PLINK v1.90 software with the following criteria: a call rate greater than 90% and a Minor Allele Frequency (MAF) greater than 0.05. Genotype imputation was carried out on the quality-controlled markers using the PHARP (V.4.0) database [11] (http://alphaindex.zju.edu.cn/PHARP/index.php/, accessed on 20 May 2026), the downstream results were interpreted with appropriate caution.

### 2.4. Estimation of Heritability

To evaluate the genetic basis of the three growth traits, genomic heritability was estimated using the Genomic Restricted Maximum Likelihood (GREML) method implemented in Genome-wide Complex Trait Analysis (GCTA) [12]. A Genomic Relationship Matrix (GRM) was constructed using quality-controlled SNP data. The following mixed linear model was fitted:

y=Xb+Zu+e

In the formula: y is the vector of phenotypic observations, b is the vector of fixed effects, u is the vector of random additive genetic effects with $u∼N(0,G\sigma_{g}^{2})$, and e is the vector of residuals with $e∼N(0,I\sigma_{e}^{2})$. X and Z are the corresponding incidence matrices.

The genomic heritability was estimated as:

$$h^{2}=\sigma_{g}^{2}/(\sigma_{g}^{2}+\sigma_{e}^{2})$$

Standard errors (SEs) of the heritability estimates were obtained directly from GCTA output.

### 2.5. Genome-Wide Association Analysis

Principal Component Analysis (PCA) was performed on the imputed SNPs using PLINK v1.90, and the results were visualized in R to assess population stratification. Subsequently, GWAS for each trait was conducted using the mixed linear model implemented in the fastGWA-MLM module of the GCTA (1.94.1) software [13]. In fastGWA, genetic relatedness among individuals was controlled by fitting a random polygenic effect based on the sparse genetic relationship matrix (sparse GRM) derived from genome-wide SNP data. Manhattan plots and Q–Q plots were generated using the CMplot (v.4.0) package. Prior to GWAS, phenotypic records were examined for completeness and plausibility. The effects of important non-genetic factors were accounted for in the analytical model. Specifically, the phenotype model included sex, rearing year and month, breed, and the first five principal components.

The MLM formula is as follows:

y=Xα+Zβ+Wμ+e

In the formula: *y* is the vector of individual phenotypes; *α* is the vector of fixed effects, including sex, rearing year and month, breed, and the first five principal components, and *X* is the design matrix for fixed effects; *β* is the vector of genotype effects, and *Z* is the genotype matrix; *μ* is the vector of random effects representing the genetic relatedness among individuals with $u∼N(0,G\sigma_{g}^{2})$, and *W* is the design matrix for random effects; e is the residual vector.

Specifically, GWAS was performed using the mixed linear model implemented in the fastGWA-MLM module of GCTA, where genetic relatedness among individuals was controlled by fitting a random effect based on the sparse genetic relationship matrix (sparse GRM). In addition, sex, rearing year and month, breed, and the first five principal components were included as fixed effects to account for non-genetic effects and population stratification.

### 2.6. Multiple Testing Correction

The genome-wide significance threshold was determined using the Bonferroni correction based on the total number of tested SNPs after quality control. Given the large number of imputed variants, this threshold was intentionally conservative to control the family-wise error rate. The model formula is as follows:

P = α/N

In the formula, P represents the significance threshold for each independent test after correction; α represents the nominal significance level for each independent test after correction; and N represents the total number of multiple hypothesis tests performed (i.e., the number of SNPs used in the GWAS analysis).

### 2.7. Annotation of Candidate Genes and Functional Enrichment Analysis

In this study, the genome-wide significance threshold was set at 0.05/N, and the suggestive significance threshold was set at 1/N, based on which significant SNP loci were screened. Candidate genes located within 500 kb upstream and downstream of the significant GWAS loci were extracted using the biomaRt package in R software, which calls the Sus scrofa 11.1 database from the Ensembl website (https://asia.ensembl.org/index.html, accessed on 9 May 2026), and were subjected to further analysis. This window was used as a practical annotation strategy rather than definitive evidence of causality. Gene Ontology (GO) and Kyoto Encyclopedia of Genes and Genomes (KEGG) enrichment analyses of the candidate genes were performed using the DAVID [14] website, and the results were visualized in R. Candidate genes were subsequently prioritized by integrating positional information with eQTL colocalization evidence, gene function, and the previous literature.

### 2.8. Colocalization Analysis

In this study, a Bayesian colocalization method was employed to identify shared causal variants between eQTLs and GWAS signals within specific genomic regions. The coloc.abffunction from the R package coloc (v5.2.3) [15] was used to perform colocalization analysis between GWASs and eQTLs for each trait, and the posterior probability (PP.H4) was calculated for each gene–tissue pair. The eQTL data were obtained from the cis-eQTL dataset of 34 tissues released by the PigGTEx project [10], including adipose tissue, the liver, muscle, and others. Colocalization regions were defined based on the significant GWAS loci. For each gene, the top SNP locus was selected from the eQTL dataset, and the region 500 kb upstream and downstream of the top SNP was extracted for colocalization analysis with GWAS. The analysis yields five posterior probabilities (PPs). In this study, a PP.H4 > 0.75 was considered to indicate the presence of a shared causal variant between GWAS and eQTL [16]. It should be noted that a high PP.H4 value indicates support for a shared association signal but does not on its own prove biological causality. Therefore, colocalization results were interpreted as supportive evidence for candidate gene prioritization.

## 3. Results

### 3.1. Statistical Analysis of Growth Traits

**Figure 1:**
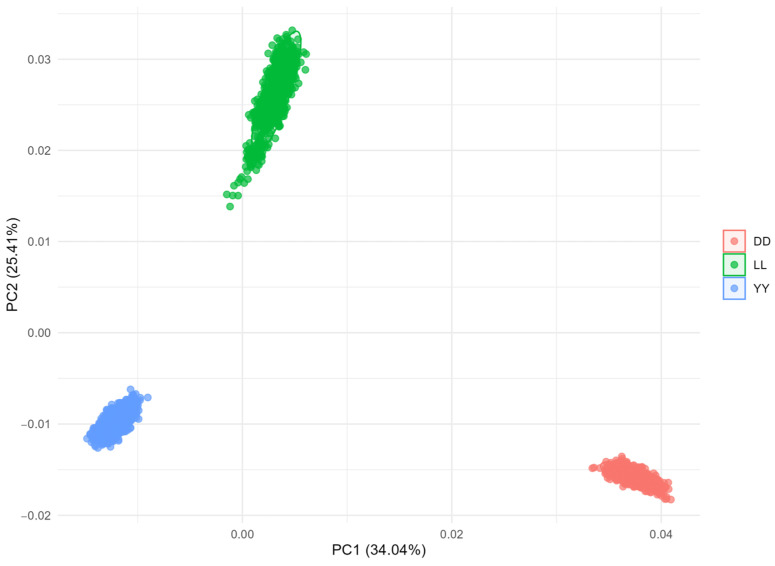
PCA plot showing the relationships among the three breeds based on the first two principal components.

Descriptive statistics for the growth traits AGE120, BF120, and LMD120 of 3363 pigs were obtained, and the results are presented in Table 4. The mean values of AGE120, BF120, and LMD120 were 171.77 days, 12.48 mm, and 67.65 mm, respectively; the genetic coefficients of variation were 8.61%, 23.31%, and 10.69%, respectively; and the heritabilities of AGE120, BF120, and LMD120 were 0.538, 0.538, and 0.424, respectively. LMD120 showed moderate heritability, while AGE120 and BF120 showed high heritability. Haplotype construction and genotype imputation analysis were performed on the 3363 pigs. After imputation, the number of SNP loci increased from 175,292 to 15,447,611 (Figure 1). The imputation accuracy was 0.98, indicating that the genotype imputation was accurate and efficient and that the imputed data can be used for further association analysis. PCA was performed on the imputed SNPs. The first two principal components, PC1 and PC2, showed a clear clustering pattern, and the PCA plot demonstrated that the three breeds were well separated (Figure 1). Because the study population consisted of multiple pig breeds, PCA was used to assess genetic structure, and the GWAS model was designed to reduce confounding from breed differentiation and relatedness. Nevertheless, as in other multi-breed GWASs, residual population stratification cannot be entirely excluded.

### 3.2. Genome-Wide Association Analysis

**Figure 2:**
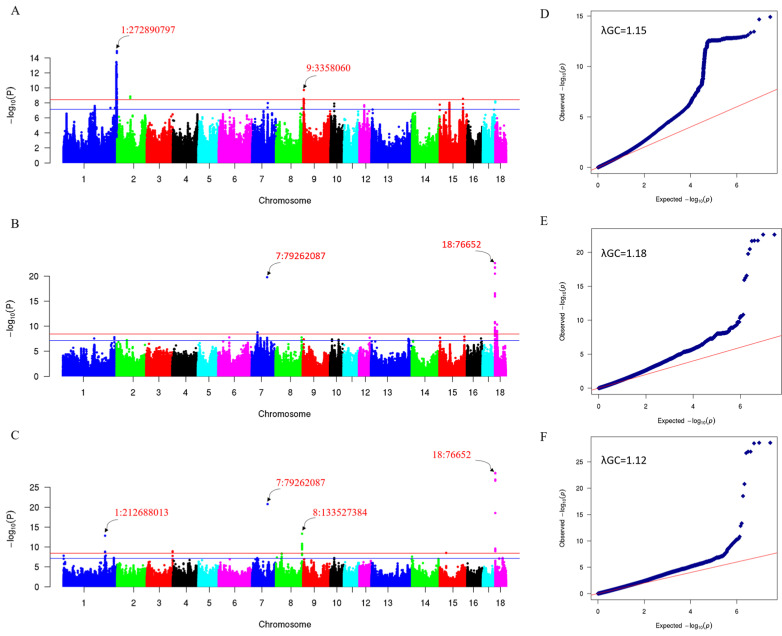
Manhattan plots of genome-wide association results for (**A**) AGE120, (**B**) BF120, and (**C**) LMD120. The red horizontal line indicates the genome-wide significance threshold based on Bonferroni correction *p* = 3.23 × 10^−9^, and the blue horizontal line indicates the suggestive significance threshold *p* = 6.47 × 10^−8^. Q-Q plots of the genome-wide association analyses for (**D**) AGE120, (**E**) BF120, and (**F**) LMD120. The *x*-axis indicates the expected $-{log}_{10}(P)$ values under the null hypothesis, and the *y*-axis indicates the observed $-{log}_{10}(P)$ values. The genomic inflation factor ($\lambda_{GC}$) for each trait is shown in the corresponding panel.

**Figure 3:**
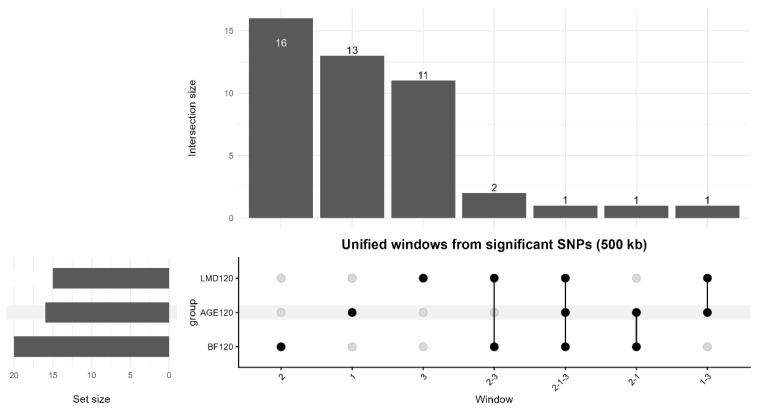
Merging of significant SNPs for each trait within a 1000 kb window. Gray dots represent QTL clusters specific to individual traits, while black dots represent the QTL cluster shared by two or three traits.

Genome-wide association analyses were performed for the AGE120, BF120, and LMD120 traits using the imputed SNPs (see Manhattan plots and Q–Q plots in Figure 2). The genomic inflation factors (λGC) for the AGE120, BF120, and LMD120 traits were 1.15, 1.18, and 1.12, respectively, indicating that systematic inflation was well controlled. As shown in the results, using the suggestive significance threshold of 1/N = 6.47 × 10^−8^, 878, 288, and 55 SNP loci were identified for AGE120, BF120, and LMD120, respectively (Supplementary Table S1). Using the genome-wide significance threshold of 0.05/N = 3.23 × 10^−9^, 434, 41, and 34 significant SNP loci were identified for AGE120, BF120, and LMD120, respectively. To further refine the candidate intervals for the significant loci, we performed LDblock analysis for the prominent SNP clusters of each trait (Supplementary Figures S1–S3). Because the Bonferroni threshold was based on the full set of imputed SNPs, it was highly stringent. This conservative criterion reduced the likelihood of false-positive findings but may also have increased false negatives, especially for loci with moderate effects. Some of the significant loci and genes located within the 500 kb upstream and downstream regions of selected significant loci are presented in Figure 3, with 45 clusters identified. Among these, 13, 16, and 11 Quantitative Trait Locus (QTL) clusters were unique to AGE120, BF120, and LMD120, respectively, while 1 QTL cluster was shared among all three traits. These QTLs were distributed on chromosomes 1, 2, 3, 6, 7, 8, 9, 10, 12, 13, 14, 15, 16, 17, and 18 (Supplementary Table S2). These genomic regions exhibit strong pleiotropy and may influence growth rate, fat deposition, and muscle development simultaneously through one or a few causal variants. A total of 422 genes were annotated within the 500 kb upstream and downstream regions of all significant loci identified in the combined-population GWAS (Supplementary Table S3). The most significant association with AGE120 was found on chromosome 1 (chr1: 272,890,797), where 27 GWAS-associated genes were detected: *DDX31*, *GTF3C4*, *AK8*, *SPACA9*, *TSC1*, *GFI1B*, *CEL*, *RALGDS*, *GBGT1*, *SURF6*, *MED22*, *RPL7A*, *SNORD24*, *SNORD36*, *SURF1*, *SURF2*, *SURF4*, *STKLD1*, *REXO4*, *ADAMTS13*, *CACFD1*, *SLC2A6*, *MYMK*, *ADAMTSL2*, *FAM163B*, *DBH*, *SARDH*, and *VAV2*. The most significant association with BF120 and LMD120 was found on chromosome 18 (chr18: 76,652), where four GWAS-associated genes were detected: *U6*, *VIPR2*, *DYNC2I1*, and *ESYT2* (Table 5).

### 3.3. GO Functional Enrichment Analysis and KEGG Pathway Enrichment

**Figure 4:**
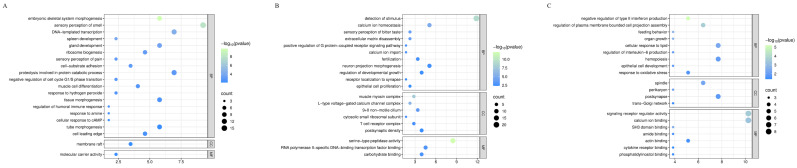
Bubble plot of GO functional enrichment for (**A**) AGE120, (**B**) BF120, and (**C**) LMD120 candidate genes. In the plots, the vertical axis represents GO terms, and the horizontal axis represents −log10(*P*). Bubble size indicates the number of enriched genes, and color intensity represents the significance level.

Within the 500 kb upstream and downstream regions of significant loci for AGE120, BF120, and LMD120, 179, 181, and 83 genes using the suggestive significance threshold of 1/N = 6.4693 × 10^−8^ were annotated, respectively. GO enrichment analysis was performed on these genes. The candidate genes for AGE120 were significantly enriched in 20 GO terms, covering biological processes, cellular components, and molecular functions (Figure 4A). The results indicate that AGE120 candidate genes are primarily involved in skeletal development, sensory perception, transcriptional regulation, immune organ development, and cell cycle regulation, which are closely associated with growth rate and overall development. The candidate genes for BF120 were significantly enriched in 20 GO terms (Figure 4B). The enrichment results suggest that these candidate genes are involved in immune regulation (interferons and IL-6), feeding behavior, organ growth, lipid response, and oxidative stress processes, which is consistent with the biological basis of fat deposition and metabolic regulation. The significantly enriched GO terms for LMD120 candidate genes are shown in Figure 4C. LMD120 is associated with lean meat percentage and loin eye area, and its candidate genes were significantly enriched in processes such as calcium ion signaling, GPCR pathways, extracellular matrix remodeling, neuronal development, and epithelial cell proliferation, suggesting that this trait may be regulated by neuro–endocrine–muscle interactions.

**Figure 5:**
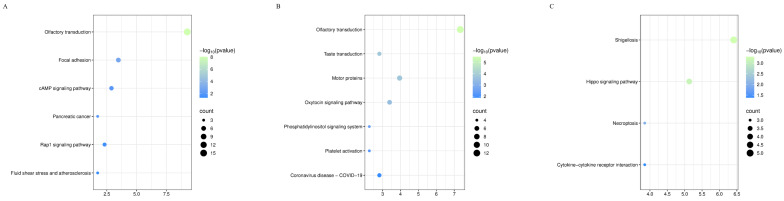
Bubble plot of KEGG pathway enrichment for (**A**) AGE120, (**B**) BF120, and (**C**) LMD120 candidate genes. The vertical axis represents pathway names, and the horizontal axis represents −log10(*P*). Bubble size indicates the number of enriched genes, and color intensity represents the significance level. Bubble size indicates the number of enriched genes, and color intensity represents the significance level.

KEGG pathway enrichment analysis was performed separately for the candidate genes of the three traits, using an adjusted *p*-value < 0.05 as the significance threshold. The candidate genes for AGE120 were significantly enriched in six KEGG pathways (Figure 5A), among which the most significant was the olfactory transduction pathway (−log10(*P*) = 7.8). These results suggest that AGE120 candidate genes may participate in cell adhesion, migration, and mechanotransduction via the cAMP and Rap1 signaling pathways, thereby influencing the overall growth rate. The candidate genes for BF120 were significantly enriched in seven KEGG pathways (Figure 5B). The most significant was also the olfactory transduction pathway (−log10(*P*) = 7.2). These enriched pathways involve neurosignal transduction (olfaction, taste, and oxytocin), cytoskeleton-regulated motor proteins, and immune responses, implying that fat deposition may be regulated by neuroendocrine and inflammatory signals. The candidate genes for LMD120 were significantly enriched in four KEGG pathways (Figure 5C). The most significant was the shigellosis pathway (−log10(*P*) = 6.8). These pathways involve cell proliferation regulation (Hippo), programmed cell death (necroptosis), and inflammatory immune responses, suggesting that this trait may be influenced by the balance between cell growth and apoptosis, as well as the immune microenvironment.

### 3.4. Colocalization Identifies Potential Genes

**Figure 6:**
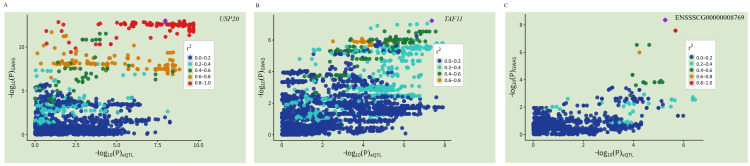
Scatter plots of GWAS and eQTL colocalization results for (**A**) AGE120, (**B**) BF120, and (**C**) LMD120.

Colocalization analysis was performed on the published significant GWAS loci and multitissue eQTL data, with a threshold of PP.H4 > 0.75. Representative scatter plots of the colocalization results are shown in Figure 6. For the AGE120 trait, a total of 13 high-confidence colocalization events (PP.H4 > 0.75) were detected, involving nine unique genes and four tissues (Table 6). The PP.H4 values for these events ranged from 0.769 to 0.971, with 10 events having PP.H4 > 0.85, indicating a high probability that the GWAS signals and eQTL signals shared causal variants. The AGE120 colocalizations were mainly distributed in the jejunum (2 events), milk (2 events), blastocyst (4 events), brain (1 event), adipose (1 event), spleen (1 event), and placenta (1 event). Among these, the blastocyst tissue contributed candidate genes supported by eQTL colocalization analysis (*CDK5RAP3*, *HOXB7*, *COPZ2*, and *SCRN2*), suggesting that embryonic development–related tissues may play a key role in the genetic regulation of AGE120.

For the BF120 trait, 11 high-confidence colocalization events (PP.H4 > 0.75) were identified, involving nine unique genes and eight tissues (Table 7). The PP.H4 values ranged from 0.777 to 0.966, with seven events having PP.H4 > 0.85. BF120 colocalizations were most abundant in muscle but were also present in other tissues, including the synovial membrane, liver, frontal cortex, heart, blood, and hypothalamus. The frequent colocalization in muscle tissue suggests that backfat thickness may be influenced by gene expression regulation in muscle, thereby affecting fat deposition.

For the LMD120 trait, only two colocalization events were detected (Table 8), but both showed high PP.H4 values (0.887 and 0.812) and shared the same locus and gene, ENSSSCG00000008769, which is currently not annotated in the Sus scrofa 11.1 assembly. These two events occurred in the ileum and liver, respectively, suggesting that this gene may be expressed in the intestine and liver and influence lean meat percentage in a tissue-specific manner. Although the function of this gene is not yet clear, its location within the QTL interval and the strong colocalization evidence make it a worthy candidate for subsequent functional validation. The number of colocalization-supported genes differed among traits. In particular, relatively fewer colocalization signals were observed for LMD120. This pattern may reflect limited statistical power of eQTL detection in trait-relevant tissues, tissue-specific regulatory architecture, or a more complex genetic basis for loin muscle depth.

### 3.5. Identification of Potential Candidate Genes

Genes within ±500 kb of the lead SNPs were first collected as positional candidates. We emphasize that this step was intended as an initial annotation procedure rather than a direct inference of causality. To improve biological interpretability, these loci were further integrated with multi-tissue PigGTEx eQTL data. Colocalization analysis identified a subset of genes with strong evidence for shared GWAS-eQTL signals, thereby increasing confidence in their candidacy. Overall, seven candidate genes were identified for AGE120 (*ZNF215*, *UBE2Z*, *HOXB7*, *SARDH*, *ADAMTSL2*, *USP20*, and *AIF1L*), nine candidate genes for BF120 (*TAF11*, *ATP6V0A4*, *RPL10A*, *ANKS1A*, *TAF11SYNGAP1*, *ZC3HAV1L*, *SYNGAP1*, and *TEAD3*), and six candidate genes for LMD120 (*NWD2*, *C4orf19*, *RELL1*, *PGM2*, *TBC1D1*, and *ssc-mir-9840*).

## 4. Discussion

In this study, we employed an integrated approach combining GWAS and expression quantitative trait locus data to identify potential causal genes for growth traits in pigs. To enhance the reliability of our findings, we adopted two independent lines of evidence. The first line considered genes located within a 500 kb window upstream and downstream of the significant GWAS loci. The second line involved the integration of eQTL data to detect overlaps between GWAS signals and eQTLs, thereby adding another layer of evidence.

Numerous studies have attempted to dissect growth traits in pigs; however, most have focused on a single trait or two traits [17], and few have investigated pigs at 120 kg body weight [18]. Ding et al. [19] and Gozalo-Marcilla et al. [20] reported nine and 64 candidate genes associated with BF, respectively. Zhuang et al. [21] and Ding et al. [22] identified eight and four potential candidate genes related to LMD, respectively. The candidate genes identified vary considerably across different study methods and genetic backgrounds; nevertheless, our study yielded results consistent with some previous findings. *ZC3HAV1L* was identified as a candidate gene for BF in another GWAS of pig growth traits [23]. *TAF11* is a subunit of the transcription factor IID (TFIID) complex and participates in RNA polymerase II–mediated transcription initiation [24]. In adipose tissue, *TAF11* may cooperate with adipogenic transcription factors such as PPARγ and C/EBPα to promote the expression of adipocyte-specific genes; the expression of *TAF11* has been associated with loin muscle depth in pig growth traits [25]. *ANKS1A* encodes ankyrin repeat and SAM domain-containing protein 1A, a multifunctional scaffold protein [26,27], and has been identified as a candidate gene closely related to growth and development in Tunchang pigs [28]. *USP20* was found to be associated with the BF growth trait in a study of 4560 pigs from three populations [29]. *TBC1D1*, a substrate of the energy sensor AMP-activated protein kinase (AMPK), is a key regulator of reactive oxygen species production in macrophages and subsequent adipose tissue inflammation; consequently, it promotes the development of obesity [30]. *TBC1D1* was also identified as a novel candidate gene in an assessment of muscle quality in Chinese Suhuai pigs [31]. In addition to identifying five genes previously reported to be associated with pig growth traits (*TAF11*, *ZC3HAV1L*, *ANKS1A*, *USP20*, and *TBC1D1*), a set of novel, high-confidence candidate genes was also identified. These genes may influence growth traits through mechanisms such as transcriptional regulation, protein degradation, myogenic–adipogenic balance pathways, signal integration, and neuroendocrine and immune regulation.

For the AGE120 trait, *ZNF215* belongs to the zinc finger protein transcription factor family, whose members typically regulate the expression of downstream target genes by binding to DNA or interacting with other proteins. Previous studies have shown that zinc finger proteins play important roles in the proliferation and differentiation of muscle stem cells [32]. *ZNF215* may be involved in regulating cell cycle progression or insulin-like growth factor signaling pathways and may thus affect the overall growth rate. This suggests that its high expression may accelerate the time required for individuals to reach 120 kg body weight. *UBE2Z* encodes ubiquitin-conjugating enzyme E2 Z, a key component of the ubiquitin–proteasome system. This system is responsible for degrading short-lived or misfolded proteins within cells, thereby regulating the cell cycle, signal transduction, and metabolic homeostasis [33]. In skeletal muscle, the ubiquitin-proteasome pathway primarily mediates protein degradation, affecting the net deposition rate of muscle proteins [34]. Genetic variants in *UBE2Z* may indirectly regulate muscle growth rate by altering ubiquitination efficiency. *HOXB7* is a member of the homeobox gene family and plays a central regulatory role in embryonic development and organ formation [35]. In adult tissues, HOX genes continue to participate in tissue homeostasis and regeneration processes [36]. Notably, *HOXB7* has been identified as a bidirectional regulator of adipogenesis and myogenic differentiation: It promotes the differentiation of preadipocytes into mature adipocytes while inhibiting the differentiation and fusion of myoblasts [37]. In fattening pigs at 120 kg, the relative proportions of muscle and adipose tissue determine the slaughter weight. High expression of *HOXB7* may promote fat deposition and inhibit muscle growth, thereby prolonging the days to 120 kg. *SARDH* encodes sarcosine dehydrogenase, a key enzyme involved in one-carbon metabolism and amino acid degradation in mitochondria, primarily catalyzing the conversion of sarcosine (N-methylglycine) to glycine [38]. In rapidly growing pigs, the efficiency of amino acid metabolic pathways directly affects protein deposition rates and feed conversion ratios. Therefore, genetic variants in *SARDH* may significantly influence growth rate by altering amino acid catabolism patterns. *ADAMTSL2* encodes ADAMTS-like protein 2, which belongs to the *ADAMTS* superfamily but lacks proteolytic activity and primarily functions as a regulator of the extracellular matrix [39,40]. *ADAMTSL2* is involved in the formation of fibrillin-1 microfibrils and elastic fiber assembly and is indispensable for connective tissue development [41]. In skeletal muscle, the structure and composition of the Extracellular Matrix affect myofiber proliferation, hypertrophy, and satellite cell migration [42]. In this study, *ADAMTSL2* may indirectly influence the overall growth rate of pigs by regulating the homeostasis of the muscle interstitial environment. *AIF1L* is a protein-coding gene localized to actin filaments and focal adhesions, where it activates actin filament binding activity. Studies have shown that deletion of the *AIF1L* protein in transgenic mice leads to reduced leptin sensitivity and circulating leptin levels [43,44]. Leptin is a hormone primarily secreted by white adipocytes that regulates food intake and energy balance in mammals [45]. *AIF1L* has been identified as a genetic modifier of leptin, influencing leptin levels, food intake, and obesity during high-fat-diet feeding. Therefore, in pigs, differential expression of *AIF1L* may affect leptin levels, thus influencing appetite and energy expenditure. This ultimately manifests as differences in feed efficiency.

For the BF120 trait, *ATP6V0A4* encodes the a4 subunit of vacuolar-type ATPase (V-ATPase) [46]. V-ATPase is responsible for pumping protons into organelles or the extracellular space to maintain an acidic environment and plays a critical role in lysosomal degradation, autophagy, endocytosis, and vesicular transport [47]. In adipocytes, autophagic flux significantly regulates lipid droplet metabolism and adipocyte size. Inhibition of V-ATPase activity blocks autophagic flux, leading to abnormal lipid accumulation [48]. Therefore, the expression level of *ATP6V0A4* may influence backfat deposition by regulating lysosomal acidification and autophagic efficiency within adipocytes. *RPL10A* encodes ribosomal protein L10a, a component of the 60S large subunit. Ribosomal proteins are not only involved in protein translation but also possess extra-ribosomal functions, such as regulating the cell cycle, DNA repair, and apoptosis [49]. During adipogenesis, an increased translation rate is necessary for preadipocyte proliferation and differentiation [50]. Differential expression of *RPL10A* may affect the hypertrophic growth of adipose tissue by modulating the efficiency of ribosomal protein synthesis.

For the LMD120 trait, although studies directly linking porcine *PGM2* to growth traits are scarce, polymorphisms in its highly homologous gene *PGAM2* (phosphoglycerate mutase 2) have been shown to be associated with growth and reproduction traits in Zhongwu pigs [51]. Given that loin muscle depth is closely related to glycolytic capacity and energy metabolism rate in skeletal muscle and that *PGM2* is located within a significant GWAS region, it is reasonable to hypothesize that variants in *PGM2* may indirectly modulate myofiber hyperplasia and hypertrophy by affecting the efficiency of glucose metabolism in muscle tissue. *RELL1* (RELT-like protein 1) has been reported to activate the p38 signaling pathway and induce apoptosis, and it is highly expressed in porcine skeletal muscle. Existing GWAS evidence in pigs has primarily associated *RELL1* with reproductive traits such as stillbirth count and mummified fetus count; however, a direct causal relationship with growth traits has not yet been established. Since loin muscle depth reflects the cumulative developmental outcome of muscle during the growing and finishing phases, the high expression of *RELL1* in skeletal muscle suggests that it may be involved in the balance between myocyte apoptosis and remodeling. If variants in *RELL1* lead to aberrant p38 activity, they might accelerate myocyte apoptosis and reduce the number of functional myofibers, thereby decreasing LMD. This hypothesis requires validation through specifically designed cellular experiments.

By employing the GWAS and eQTL colocalization strategy, this study successfully focused association signals onto specific genes, thereby avoiding the false-positive issues that arise from relying solely on physical position annotation. The identified candidate genes span multiple biological levels, including metabolism, development, translation, and degradation, providing new entry points for a deeper understanding of the molecular mechanisms underlying pig growth traits at the 120 kg stage. These genes can be further utilized to develop molecular markers to assist genomic selection in breeding.

This study has several limitations that should be acknowledged. First, although population structure and relatedness were accounted for in the GWAS model, the use of a combined multi-breed population may still introduce residual confounding due to breed-specific genetic backgrounds. Second, candidate genes were initially annotated based on physical proximity to significant loci, which may not always identify the true causal genes. Third, although eQTL colocalization provides supportive evidence for shared regulatory signals, it does not establish causality without further fine-mapping, conditional analyses, or experimental validation. Fourth, the stringent Bonferroni correction based on dense imputed variants may have increased false negatives. Finally, the identified loci and candidate genes were not independently replicated in another population, and no functional experiments were conducted. Therefore, the results should be interpreted as prioritizing candidate genes and genomic regions for future validation rather than providing definitive causal conclusions.

## 5. Conclusions

In summary, by integrating GWASs with multi-tissue eQTL colocalization analysis, this study prioritized a series of candidate genes associated with growth traits in pigs at the 120 kg stage. In addition to previously reported genes such as *TAF11*, *ZC3HAV1L*, *ANKS1A*, *USP20*, and *TBC1D1*, we identified several novel high-confidence candidate genes, including *ZNF215*, *UBE2Z*, *HOXB7*, *SARDH*, *ADAMTSL2*, *ATP6V0A4*, *RPL10A*, *PGM2*, and *RELL1*. These genes may contribute to variation in growth-related phenotypes through regulatory, metabolic, developmental, translational, and signaling pathways. Overall, our findings expand the current understanding of the genetic architecture of pig growth traits and provide a useful foundation for future functional validation and molecular breeding applications.

## Figures and Tables

**Table 4:** Descriptive statistics and genomic heritability estimates of growth traits in pigs.

Trait	N	Mean	SD	Max	Min	CV/%	h2	SE
AGE120	3363	171.77	14.79	260.39	134.23	8.61	0.538	0.2551
BF120	3363	12.48	2.90	25.56	6.24	23.21	0.538	0.051
LMD120	3232	67.65	7.23	94.53	15.91	10.69	0.424	0.1274

**Table 5:** Candidate genes identified near significant GWAS loci.

Trait	Single Nucleotide Polymorphism	Standard Error	*p*-Value	Gene Name
AGE120	1:270551699	0.614738	2.04 × 10^−13^	*USP20*, *FNBP1*, *GPR107*, *NCS1*, *HMCN2*, *ASS1*, *FUBP3*, *PRDM12*, *EXOSC2*, *ABL1*, *QRFP*, *FIBCD1*, *LAMC3*
	1:270552196	0.617713	3.71 × 10^−13^	
	1:270552202	0.617904	4.54 × 10^−13^	
	1:270552204	0.617904	4.54 × 10^−13^	
	1:270552300	0.617521	3.70 × 10^−13^	
	1:270554330	0.615725	2.21 × 10^−13^	
	1:270555026	0.650279	2.89 × 10^−13^	
	1:270555055	0.650279	2.89 × 10^−13^	
	1:270555155	0.645461	1.26 × 10^−13^	
	1:270555162	0.648068	1.33 × 10^−13^	
BF120	18:76652	0.153772	2.47 × 10^−23^	*U6*, *VIPR2*, *DYNC2I1*, *ESYT2*
	18:76654	0.153772	2.47 × 10^−23^	
	18:76678	0.154656	1.88 × 10^−22^	
	18:76680	0.154656	1.88 × 10^−22^	
	18:76646	0.152787	2.19 × 10^−22^	
	18:76670	0.148266	3.40 × 10^−21^	
	18:77895	0.12012	2.79 × 10^−17^	
	18:77891	0.120213	5.92 × 10^−17^	
	18:77918	0.120057	1.21 × 10^−16^	
LMD120	18:76652	0.381088	2.34 × 10^−29^	*U6*, *VIPR2*, *DYNC2I1*, *ESYT2*
	18:76654	0.381088	2.34 × 10^−29^	
	18:76646	0.378625	3.02 × 10^−29^	
	18:76678	0.383299	1.17 × 10^−27^	
	18:76680	0.383299	1.17 × 10^−27^	
	18:76670	0.367324	2.10 × 10^−27^	
	18:76671	0.343458	3.00 × 10^−19^	

**Table 6:** Colocalization results of GWAS and eQTL for AGE120.

Tissue	Gene Name	n_snp	pp_h0	pp_h1	pp_h2	pp_h3	pp_h4
Jejunum	*ZNF215*	2429	8.51 × 10^−7^	0.001085	2.25 × 10^−5^	0.027731	0.971161267
Milk	*ATP5G1*	1480	7.90 × 10^−10^	2.34 × 10^−7^	0.000205	0.05991	0.939884179
Milk	*UBE2Z*	1480	1.01 × 10^−6^	0.000299	0.000215	0.062947	0.936536832
Blastocyst	*CDK5RAP3*	821	6.73 × 10^−12^	2.01 × 10^−9^	0.000246	0.072525	0.927229955
Blastocyst	*HOXB7*	1476	6.11 × 10^−5^	0.018113	0.00032	0.093923	0.887582503
Brain	*SARDH*	2468	0.00058	0.038756	0.001286	0.085048	0.874330816
Adipose	*PNPO*	821	0.000264	0.079061	0.000201	0.059291	0.861182221
Blastocyst	*COPZ2*	821	3.31 × 10^−5^	0.009887	0.000482	0.143243	0.846355173
Jejunum	*ADAMTSL2*	2207	0.00159	0.105868	0.000994	0.06533	0.826217653
Blastocyst	*SCRN2*	463	5.95 × 10^−9^	3.76 × 10^−8^	0.027295	0.171749	0.800955241
Spleen	*USP20*	2462	6.79 × 10^−14^	1.79 × 10^−5^	7.63 × 10^−10^	0.200556	0.799425755
Placenta	*AIF1L*	3049	0.000325	0.03747	0.001667	0.19115	0.769388236

**Table 7:** Colocalization results of GWAS and eQTL for BF120.

Tissue	Gene Name	n_snp	pp_h0	pp_h1	pp_h2	pp_h3	pp_h4
Muscle	*TAF11*	3135	1.52 × 10^−23^	6.25 × 10^−21^	8.54 × 10^−5^	0.034255	0.96566
Synovial_membrane	*MRRF*	32	1.43 × 10^−9^	3.87 × 10^−10^	0.03731	0.009151	0.953539
Muscle	*ATP6V0A4*	316	0.006514	0.006148	0.0179	0.015939	0.953499
Muscle	*RPL10A*	1235	1.02 × 10^−7^	9.24 × 10^−6^	0.000754	0.067462	0.931775
Liver	*ANKS1A*	3463	9.62 × 10^−7^	0.0004	0.000301	0.124019	0.875279
Muscle	*TAF11*	3619	3.28 × 10^−34^	1.00 × 10^−31^	0.000428	0.130398	0.869174
Frontal_cortex	*SYNGAP1*	3893	9.31 × 10^−6^	0.006312	0.000219	0.147634	0.845826
Muscle	*ZC3HAV1L*	316	0.012847	0.012124	0.067292	0.062659	0.845077
Heart	*TAF11*	3207	8.95 × 10^−6^	0.003704	0.000523	0.215515	0.780249
Blood	*PIGN*	11	0.192843	0.013941	0.014413	0.000263	0.77854
Hypothalamus	*TEAD3*	1148	0.00174	0.160359	0.000659	0.059917	0.777325

**Table 8:** Colocalization results of GWAS and eQTL for LMD120.

Tissue	Gene_Id	n_snp	pp_h0	pp_h1	pp_h2	pp_h3	pp_h4
Ileum	ENSSSCG00000008769	1004	0.000152	0.040267	0.000278	0.072728	0.886574
Liver	ENSSSCG00000008769	1062	0.000513	0.135802	0.000198	0.051639	0.811848

## Data Availability

Upon reasonable request, the datasets of this study can be available from the corresponding authors.

## Supplementary Materials

The following supporting information can be downloaded at: https://www.mdpi.com/article/10.3390/biology15141216/s1, Table S1: All significant SNPs for AGE120, BF120 and LMD120; Table S2: Unified_windows_500kb for AGE120, BF120 and LMD120; Table S3: Genes annotated within 500 kb upstream and downstream of the significant loci for AGE120, BF120 and LMD120; Figure S1: LD heatmap of a critical spanning from 270551699 to 270654149 on chromosome 1 for AGE120; Figure S2: LD heatmap of a critical spanning from 10683054 to 10763165 on chromosome 18 for BF120; Figure S3: LD heatmap of a critical spanning from 76633 to 77918 on chromosome 18 for LMD120.
