# Supplementary figures and images for: Integrated GWAS and eQTL Colocalization Identified Candidate Genes for Growth Traits in Pigs
Source: Biology (Basel). 2026 Jul 22;15(14):1216. doi: 10.3390/biology15141216 (PMC13403683; doi:10.3390/biology15141216)

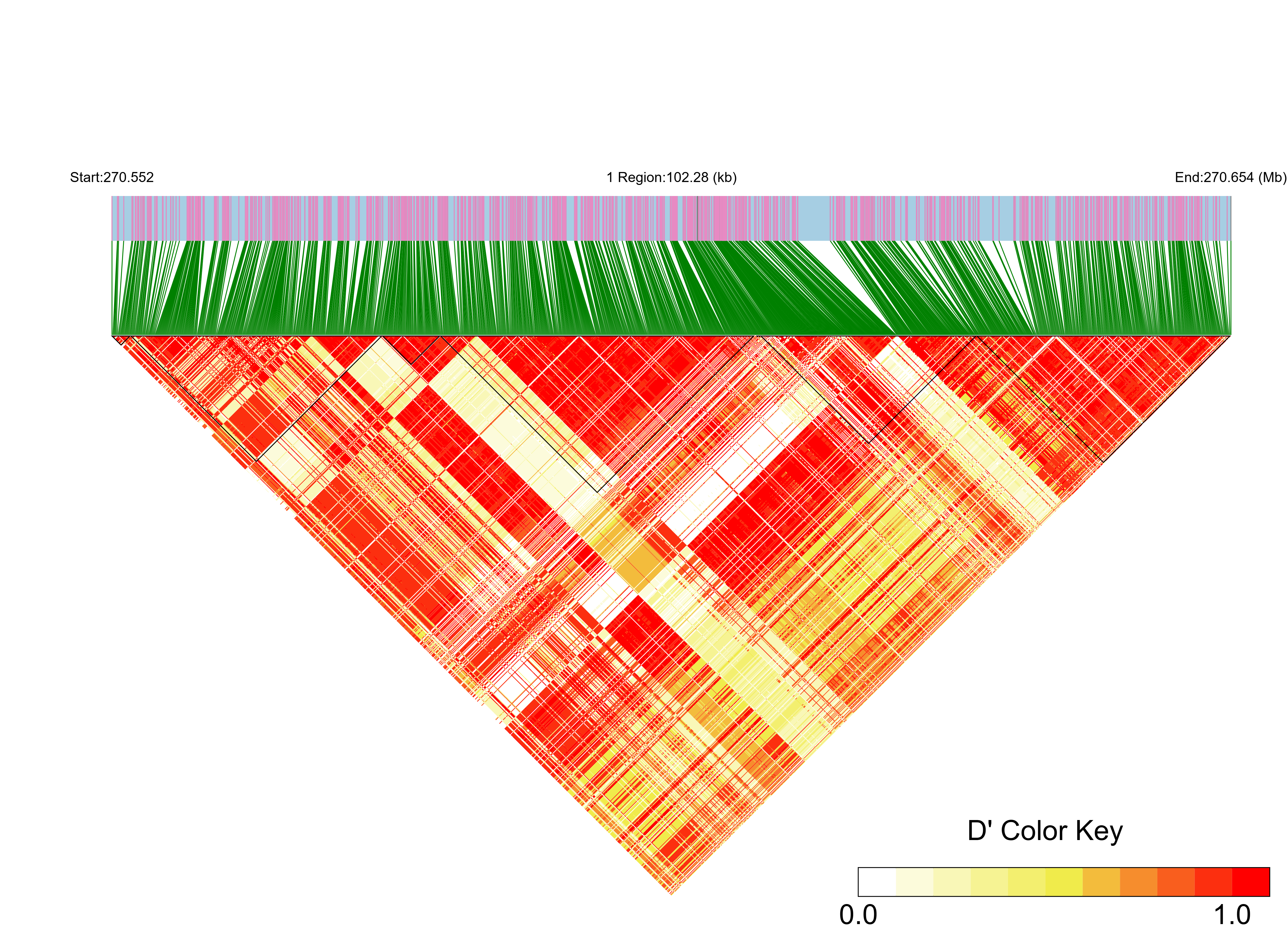

Supplement: Supplementary file 1 [file biology-15-01216-s001.zip › Supplementary Materials/Figure S1 LD heatmap of a critical spanning from 270551699 to 270654149 on chromosome 1 for AGE120.png]

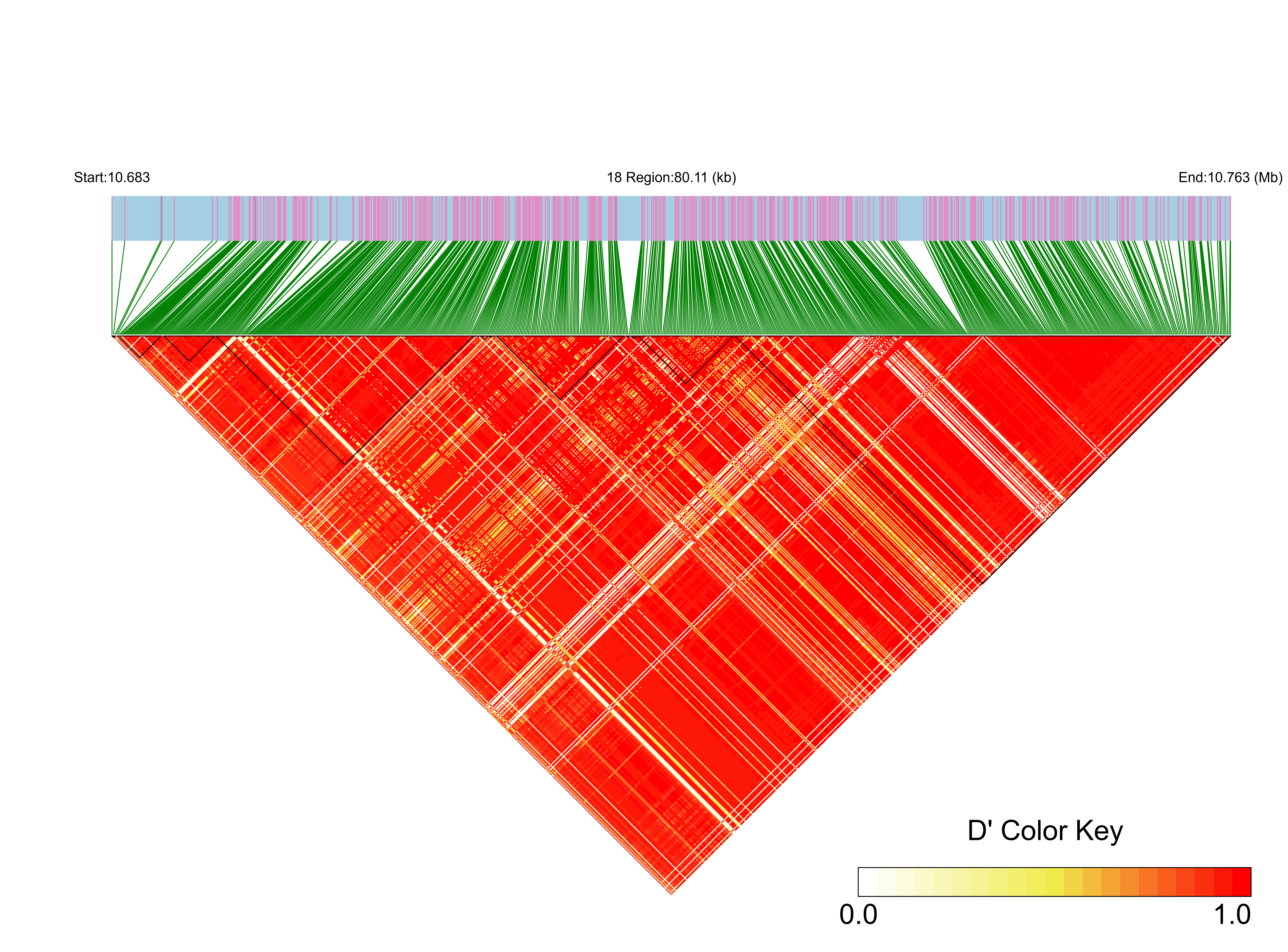

Supplement: Supplementary file 1 [file biology-15-01216-s001.zip › Supplementary Materials/Figure S2 LD heatmap of a critical spanning from 10683054 to 10763165 on chromosome 18 for BF120.png]

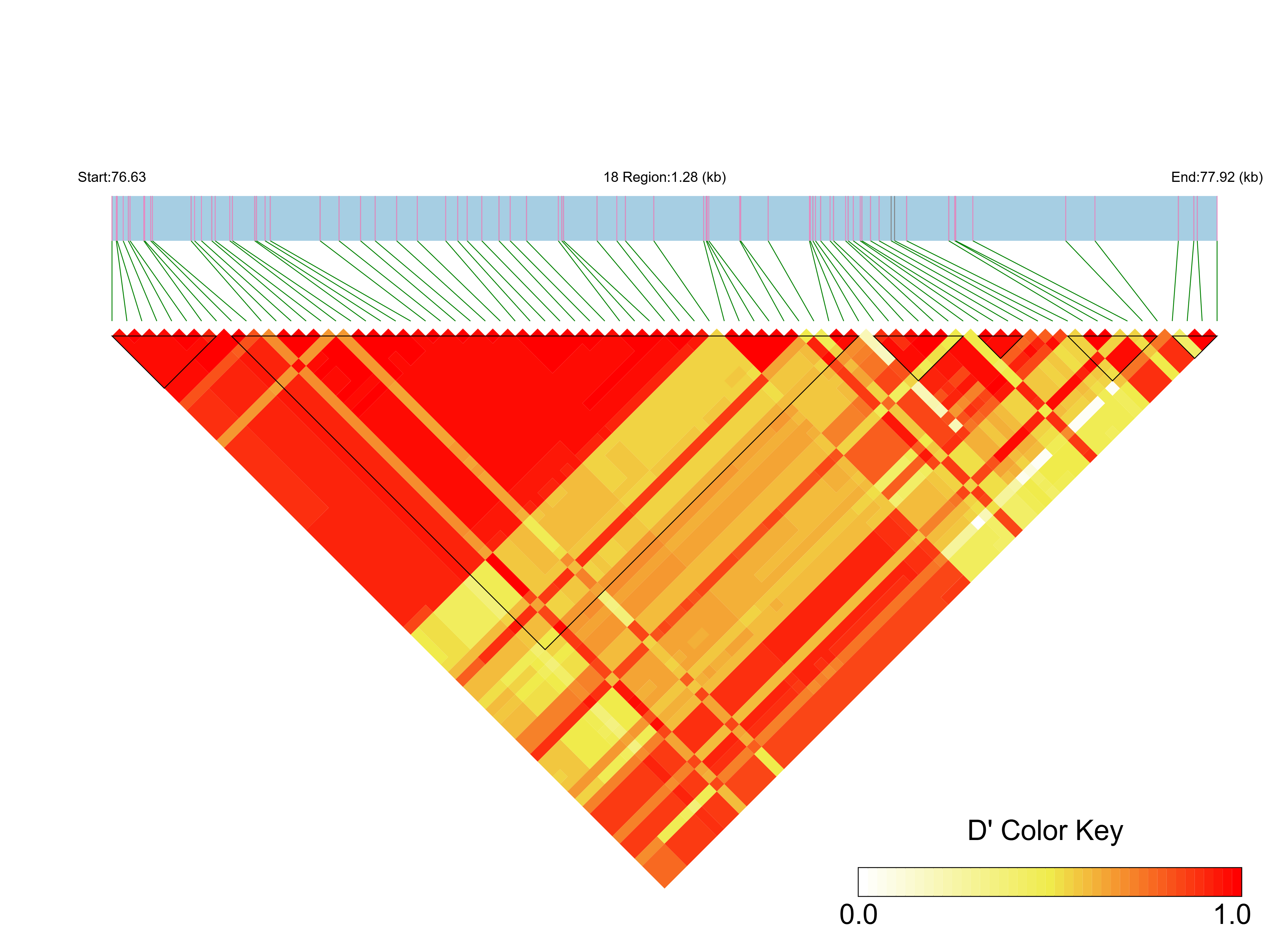

Supplement: Supplementary file 1 [file biology-15-01216-s001.zip › Supplementary Materials/Figure S3 LD heatmap of a critical spanning from 76633 to 77918 on chromosome 18 for LMD120.png]
